# Single-cell transcriptome analysis reveals the metabolic changes and the prognostic value of malignant hepatocyte subpopulations and predict new therapeutic agents for hepatocellular carcinoma

**DOI:** 10.3389/fonc.2023.1104262

**Published:** 2023-01-31

**Authors:** Cuifang Han, Jiaru Chen, Jing Huang, Riting Zhu, Jincheng Zeng, Hongbing Yu, Zhiwei He

**Affiliations:** ^1^ Guangdong Provincial Key Laboratory of Medical Molecular Diagnostics, The First Dongguan Affiliated Hospital, Guangdong Medical University, Dongguan, China; ^2^ School of Pharmacy, Guangdong Medical University, Dongguan, China; ^3^ Dongguan Key Laboratory of Medical Bioactive Molecular Developmental and Translational Research, Guangdong Provincial Key Laboratory of Medical Molecular Diagnostics, Guangdong Medical University, Dongguan, China

**Keywords:** cancer metabolism, hepatocellular carcinoma, malignant hepatocytes, prognostic biomarker, single-cell RNA sequencing

## Abstract

**Background:**

The development of HCC is often associated with extensive metabolic disturbances. Single cell RNA sequencing (scRNA-seq) provides a better understanding of cellular behavior in the context of complex tumor microenvironments by analyzing individual cell populations.

**Methods:**

The Cancer Genome Atlas (TCGA) and Gene Expression Omnibus (GEO) data was employed to investigate the metabolic pathways in HCC. Principal component analysis (PCA) and uniform manifold approximation and projection (UMAP) analysis were applied to identify six cell subpopulations, namely, T/NK cells, hepatocytes, macrophages, endothelial cells, fibroblasts, and B cells. The gene set enrichment analysis (GSEA) was performed to explore the existence of pathway heterogeneity across different cell subpopulations. Univariate Cox analysis was used to screen genes differentially related to The Overall Survival in TCGA-LIHC patients based on scRNA-seq and bulk RNA-seq datasets, and LASSO analysis was used to select significant predictors for incorporation into multivariate Cox regression. Connectivity Map (CMap) was applied to analysis drug sensitivity of risk models and targeting of potential compounds in high risk groups.

**Results:**

Analysis of TCGA-LIHC survival data revealed the molecular markers associated with HCC prognosis, including MARCKSL1, SPP1, BSG, CCT3, LAGE3, KPNA2, SF3B4, GTPBP4, PON1, CFHR3, and CYP2C9. The RNA expression of 11 prognosis-related differentially expressed genes (DEGs) in normal human hepatocyte cell line MIHA and HCC cell lines HCC-LM3 and HepG2 were compared by qPCR. Higher KPNA2, LAGE3, SF3B4, CCT3 and GTPBP4 protein expression and lower CYP2C9 and PON1 protein expression in HCC tissues from Gene Expression Profiling Interactive Analysis (GEPIA) and Human Protein Atlas (HPA) databases. The results of target compound screening of risk model showed that mercaptopurine is a potential anti-HCC drug.

**Conclusion:**

The prognostic genes associated with glucose and lipid metabolic changes in a hepatocyte subpopulation and comparison of liver malignancy cells to normal liver cells may provide insight into the metabolic characteristics of HCC and the potential prognostic biomarkers of tumor-related genes and contribute to developing new treatment strategies for individuals.

## 1 Introduction

The mortality rate for liver cancer is the third highest among all cancers, and it is the sixth most frequent cancer overall (1). Hepatocellular carcinoma (HCC) is a tumour of hepatocellular origin. HCC is the predominant pathological type of primary liver cancer (PLC), as it represents 75-85% of all instances of PLC (2). A vast majority of HCCs are caused by chronic disease, and most of these cases reportedly evolve from chronic liver disease. This is primarily because of viral infections, including hepatitis B virus (HBV) and hepatitis C virus (HCV), and alcohol misuse (3). It is recommended that patients diagnosed with HCC in the early stages receive surgical resection, liver transplantation, and local resection (radiofrequency ablation) according to the Barcelona Clinic Liver Cancer (BCLC) staging system. Those in the intermediate stage are widely treated with trans-arterial chemoembolization (TACE), whereas systemic therapies are mainly considered for advanced-stage patients. Advanced-stage patients are often symptomatic, although they exhibit some degree of impaired liver function (4, 5). Notably, few or no treatments are available to improve survival rates for patients in advanced stages.

The development of treatment modalities for advanced HCC has dramatically expanded recently. To date, the FDA has approved several oral tyrosine kinase inhibitors (lenvatinib, regorafenib and cabozantinib), immune checkpoint inhibitors (nivolumab and pembrolizumab) and immunotherapies, such as monoclonal antibodies (6–8). These therapies have steadily improved the overall survival (OS) of HCC patients. However, the prognosis for HCC patients continues to be poor because of recurrence and elevated metastasis rates (9). HCC features have been attributed to a small subpopulation of tumour cells that carry more aggressive genetic or phenotypic alterations that allow them to escape conventional detection methods (10).

Although conventional bulk RNA sequencing (bulk RNA-seq) can provide sufficient gene expression profiles of large blocks of tissue, it does not effectively distinguish between different cell lineages and cellular interactions (11). Recently, the emergence of single-cell sequencing technology has bridged the gap between traditional high-throughput sequencing technologies and microarray data to provide genomic, transcriptomic, and epigenetic information from individual cells (12). Tumours consist of three major cell types, namely, malignant, immune and stromal cells, whose spatiotemporal interactions constitute a complex ecosystem (13). Unravelling the interactions between these types involves understanding tumour development and prognosis and therapeutic options. Since the advent of single-cell sequencing, various researchers have produced a relatively complete picture of human cell atlas, which has subsequently provided a great reference for understanding the complex composition of the organs of the body (14). Additionally, single-cell sequencing has been extensively employed to reveal the molecular mechanisms underlying HCC. For instance, studies have mapped the single-cell landscape of the early recurrent HCC ecosystem by relying on the high recurrence and low survival rates of HCC patients to advance the immunotherapy guidelines for HCC (13). Numerous studies have utilized single-cell sequencing techniques to elucidate the heterogeneity of malignant tumour cells, stromal cells, and immune cells. The large scale single-cell omics study targeting tumor-associated T cells published by Zhang et al. sketched the tumor immune landscape and laid the groundwork for a multifaceted understanding of T-cell characteristics associated with liver cancer (15). Single-cell technology can also identify rare subpopulations that were previously undetected by bulk RNA sequencing techniques, and these cell types are pivotal in determining tumor characteristics, including stemness-associated malignant cells and cancer-associated fibroblasts (16–18).

The reprogramming of energy metabolism characterizes tumour cells and causes rapid cell growth and proliferation. Thus, it is one of the hallmarks of cancer. Tumour cells actively take up glucose through the uncommon process of anaerobic glycolysis (Warburg effect). Studies have shown that this process provides energy to tumour cells, permitting intermediates to enter the anabolic bypass to maintain the *de novo* synthesis of nucleotides, lipids, and amino acids needed for cell proliferation (19). HCC is closely linked to metabolic abnormalities, as the liver is the primary metabolic organ. Most previous studies concerned with liver cancer have focused on sequencing at the tissue level to reveal the overall metabolic alterations. Single-cell sequencing technology can compensate for the shortcomings of bulk sequencing, thereby allowing one to pinpoint the cell groups most significantly associated with metabolic alterations from a large number of cell types. This also allows researchers to comprehensively describe the overall changes in gene expression patterns and reveal changes across specific cell groups. Therefore, scRNA-seq and bulk RNA-seq integration are important techniques for studying tumour development and heterogeneity. We analysed published single-cell transcriptome sequencing data to identify metabolically relevant HCC subpopulations, namely, hepatic epithelial cells. We then used the identified differentially expressed genes to designate a prognostic model for HCC patients.

## 2 Materials and methods

### 2.1 Data collection

The scRNA-seq data for HCC patients were acquired from GEO(https://www.ncbi.nlm.nih.gov/geo/, accession number GSE149614) and TCGA (https://portal.gdc.cancer.gov/) databases, respectively. TCGA-LIHC samples with complete clinical information were utilized as the model training set, and HCC samples from the GEO database (GSE76427) were utilized as the external validation set.

We first constructed a human liver cell atlas by performing cell classification and marker gene identification relying on Seurat. There were 17 samples in total from 10 HCC patients. These included 8 tumour samples (PT), 8 normal paraneoplastic samples (NTL), and one metastatic lymph node sample (MLN). The data for these samples were obtained from the GSE149614 project.

### 2.2 Identification of HCC cell subtypes

The scRNA-seq data were assessed by the Seurat package implemented in R software (4.1.1), with the exclusion of samples with more than 30% mitochondrial genes. The data were normalized using the Normalize Data function, and 2,000 genes with high intercellular coefficients of variation were subsequently extracted. Principal component analysis (PCA) was then performed, with 15 PCs selected for subsequent uniform manifold approximation and projection (UMAP) analysis. Cell types within the obtained clusters were annotated by the reported cell marker genes, and the expression matrix was generated for further analysis.

### 2.3 Analysis of intercellular communications

To investigate the potential interactions between tumor and paracancerous normal HCC samples, we employed the CellChat (1.5.0) package to analyse intercellular communication. We performed CellChat analysis of the annotated cellular gene expression profile data according to the official workflow. This package mimics intercellular communication by assessing the binding ligands and receptors along with their cofactors (20). Depending on receptor expression in one cell type and ligand expression in the other, enriched receptor−ligand interactions between the two cell types were inferred. Signaling pathways were visualized using the “netVisual_aggregate” function, where ligands were defined as efferent signals and receptors were defined as afferent signals.

### 2.4 Identification of important metabolic pathways at the single-cell level

Next, we employed the ‘scMetabolism’ package (0.2.1) to calculate the metabolic state between different cell types in the HCC dataset. This package combines published gene sets from the Kyoto Encyclopedia of Genes and Genomes (KEGG) database and the Reactome database to easily quantify single-cell metabolic activity. (21). Here, we used the authors’ integrated list of metabolism-related gene sets from the Reactome database to explore metabolic pathway changes among six cell subpopulations and further looked at metabolic changes in epithelial cell subpopulations between tumor and paracancerous normal HCC samples.

### 2.5 Copy number variation analysis

To identify malignant cells in HCC patients, we compared patterns of chromosomal gene expression across cancer cells to those of their putative noncancerous counterparts using the infercnv package (version 1.12.0). First, we downloaded the human genome annotation file from the gencode database (https://www.gencodegenes.org/human/), converting it into a genomic location file. We used paracancerous epithelial tissue expression profiles from HCC patients as a reference group. Because our data were 10x scRNA-seq data, we set 0.1 as the cut-off value, and the denoise = T. Referring to the two indicators used by Itay Tirosh et al. to determine benign versus malignant cells, here we used the overall copy number variant (CNV) and the correlation with the average CNV of the top 5% of cells from the same tumor to estimate the malignancy or non-malignancy of the cells (22). The following correlation reference thresholds for determining the malignancy or not of cells were given: malignant cells: overall CNV > 0.2 & CNV correlation of the top 5% of tumors > 0.2; non-malignant cells: overall CNV < 0.2 & CNV correlation of the top 5% of tumors < 0.2.

### 2.6 Identification of significantly related pathways across different epithelial cell types

After scoring individual cells using a variety of enrichment methods, we derived multiple gene set enrichment score matrixes using the 'irGSEA' package (https://github.com/chuiqin/irGSEA/). Next, we calculated the differentially expressed gene sets for every single cell subpopulation within the enrichment score matrix for every gene set using the Wilcoxon test. Employing heat maps, certain specific enrichment pathways were labelled and then visualized.

### 2.7 Generation and validation of prognostic features

Univariate Cox analysis was used to screen genes associated with OS in TCGA-LIHC patients based on scRNA-seq and bulk RNA-seq datasets, and then, LASSO analysis was used to select significant predictors for incorporation into multivariate Cox regression. Next, we selected and used prognostic characteristics to generate polygenic risk scores and stratify TCGA-LIHC samples into either low- or high-risk groups. We also generated time-dependent receiver operating characteristic (ROC) curves to assess the predictive power of the prognostic features. The GSE76427 dataset was used to validate the prognostic value of the prognostic features. The entire analysis and visualization processes were performed by the survival, survminer, rms, and time ROC packages in R.

### 2.8 Gene expression of prognostic genes

Total RNA from cells was extracted with TRIzol reagent (Thermo Fisher Scientific, 15596026) following the manufacturer’s instructions. Complementary DNA (cDNA) was synthesized and PCRs with cDNA as template were performed using a real-time detector (The Applied Biosystems QuantStudio 5 Real-Time PCR System) using Hieff qPCR SYBR Green Master Mix. The primer sequences are shown in **Supplementary Table S1**. Transcript levels were normalized against beta-actin levels as an internal reference and were evaluated using the 2- Δ ΔCt method. All experiments were repeated three times.

The Human Protein Atlas (HPA) tool was used to visually display the protein expression of prognostic genes in the form of immunohistochemical staining. The Gene Expression Profiling Interaction Analysis (GEPIA) database was applied to further demonstrate the credibility of the results.

### 2.9 Cell culture and western blot

An immortalized nontumorigenic normal human hepatocyte cell line MIHA and HCC cell lines HCC-LM3 and HepG2 were purchased from the Fenghui Biotech Co., Ltd. (Hunan, China) with STR report. The MIHA cells were cultured in RPMI-1640 and HCC-LM3 and HepG2 were cultured in Dulbecco’s modified Eagle medium (Gibco, Gaithersburg, MD, USA) with 10% fetal bovine serum (FBS, Sigma), 100 μg/mL penicillin and 100 μg/mL streptomycin (Solarbio, Shanghai, China) at 37°CC and 5% CO^2^.

Total protein was extracted by using Takara kit. The Protein concentration was detected by BCA assay. The primary antibodies used in this study were anti-CYP2C9 (1:1000, Abcam), anti-PON1(1:1000, Abcam) anti-beta-Actin (1:1000, Cell Signaling Technology).

### 2.10 Drug sensitivity analysis

Genomics of Drug Sensitivity in Cancer (GDSC, https://www.cancerrxgene.org/) is the largest pharmacogenomic database that is freely accessible for predicting responses to anticancer drugs. GDSC comprises 2 databases, namely, GDSC1, which contains 958 cell lines and 367 drugs, and GDSC2, which contains 805 cell lines and 198 drugs (23). To explore the differences in drug treatment effects among HCC patients, drug inferred sensitivity scores were assessed in GDSC2 by the ‘oncoPredict’ package.

### 2.11 Connective map analysis

The Connective Map (CMap) database stores a large-scale resource of expression profile data of cell lines under different drug treatments, which allows rapid targeting of drug candidates for the treatment of target diseases based on aberrant transcriptomic features in tumor cells (24). These drugs have an inverse relationship with tumor-promoting factors and may regulate aberrantly expressed genes in the opposite direction.

Recently, Yang et al. used the Library of Integrated Network-based Cellular Signatures (LINCS) database to demonstrate that using the eXtreme Sum (XSum) algorithm is most likely to yield optimal results in matching compounds and disease features, demonstrating better drug retrieval performance than the other five available methods, and obtaining practical targets with desirable results in liver cancer (25). In addition, the parameters for achieving the best prediction performance in this study were set at a number of disease molecular features of 100. Considering the significant difference in dimensionality between CMap data and LINCS, we incorporate more query signatures using top300 genes for XSum analysis for potential drug prediction.

### 2.12 Statistical analysis

All statistical analyses were carried out using packages implemented in R version 4.2.0 (https://www.r-project.org/). Student’s t test was used to perform comparisons of continuous variables between two groups, and the Wilcoxon rank sum test was used to compare more than two groups. Kaplan−Meier curves with log-rank statistics were used to compare differences in OS between the two groups. Statistical significance was represented by p < 0.05.

## 3 Results

### 3.1 Single-cell gene expression profiles reveal six major cell types in the TME of primary HCC tumours

**Figure 1:**
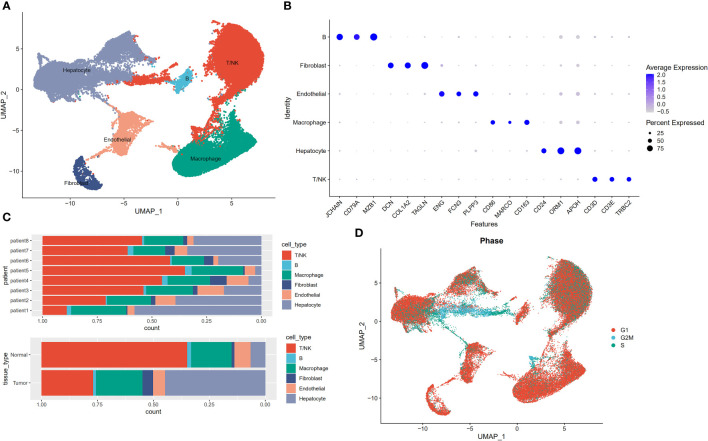
Profiles of single cells isolated from 8 primary liver cancer lesions with matching adjacent samples. **(A)** Uniform manifold approximation and projection (UMAP) plot of the analysed single cells. Each colour reflects one cell type. **(B)** Expression of marker genes for Hepatocytes, Macrophage cells, Endothelial cells, Fibroblasts, Mast cells, B cells, and T/NK cells. **(C)** Distribution of cells derived from either different patients or different sample origins. **(D)** UMAP clustering of 58,475 cells. Every colour represents a distinct cell cycle stage.

We performed descending and unsupervised cell clustering to recognize cell types based on their expression profiles. The raw dataset was read using the Seurat package. Then, an initial screening of genes and cells was performed using the following criteria: a gene had to be expressed in at least 3 cells, and at least 200 genes were measured in this cell. This was followed by further quality control to extract cells with >200 and <8000 expressed genes and <30% of mitochondrial genes. Next, the data were normalized to obtain 2000 highly variable genes for subsequent downscaling. Removal of the cell cycle effect resulted in an expression matrix comprising 58,475 cells and 24,746 genes. Next, we employed known marker genes to define broad cell categories and obtained the following six major cell subpopulations: T/NK cells, hepatocytes, macrophages, endothelial cells, fibroblasts, and B cells (**Figures 1A, B**). Cells from tumours and normal paracancerous tissues from different patients were classified into six categories (**Figure 1C**). Because proliferation is a hallmark of tumour cells, we employed the cell cycle scoring method to analyse the cell cycle. This image shows the results indicated that most of the cells were in the G1 phase, and a small number of cells were in the G2/M and S phases (**Figure 1D**).

### 3.2 Genes associated with the glucose and lipid metabolic pathway are upregulated in hepatocytes

**Figure 2:**
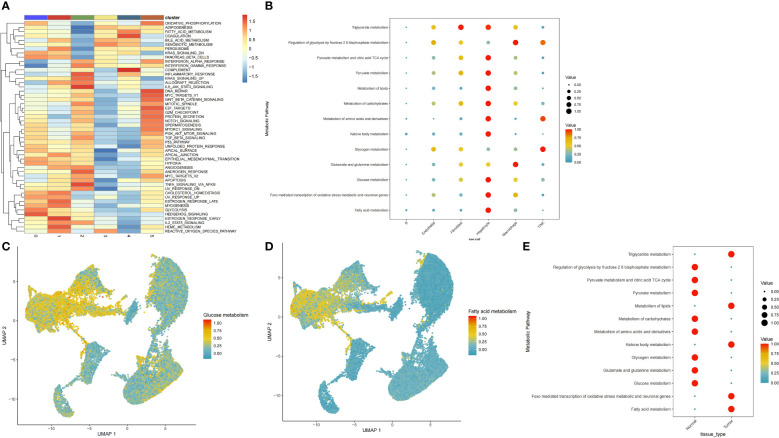
Distribution of glucose and lipid metabolic pathways in cellular subpopulations. **(A)** Functional annotation of six cellular subpopulations. **(B)** Dot plots show the specific metabolic pathways that were enriched in each cell subpopulation. **(C, D)** Scatter plots demonstrating highly expressed glucose and lipid metabolic pathways in hepatocytes cells. **(E)** Metabolic pathways comparison in hepatocytes cells from tumour versus paracancerous tissue.

To explore the existence of pathway heterogeneity across different cell subpopulations, we performed pathway activity and GSEA using signature genomes. Numerous pathways associated with cancer were upregulated in the hepatocyte subpopulation; these pathways included oxidative phosphorylation, glycolysis, and the metabolism of fatty acids, bile acids, and xenobiotics (**Figure 2A**). Next, we used the scMetabolism package to calculate scores for each metabolic pathway in each cell. We found that the epithelial cell subpopulation was enriched in most metabolic pathways, mainly those regulating pyruvate metabolism, the citrate tricarboxylic acid cycle, and the metabolism of triglycerides, pyruvates, lipids, carbohydrates, amino acids and their derivatives, ketone bodies, glucose, and fatty acids, and FoxO-mediated oxidative stress (**Figure 2B**). The genes of glucose metabolism and lipid metabolism pathways were also upregulated in epithelial cells (**Figures 2C, D**). To determine the differences in metabolic pathways of hepatic epithelial cells between tumor and paracancerous tissues, we extracted a separate subpopulation of hepatocytes and analysed the enrichment of metabolic pathways. Strikingly, we found an opposite trend between the glucose metabolism and lipid metabolism pathways in tumour and paracancerous cells (**Figure 2E**). Consequently, we subjected this cell subpopulation to more in-depth analysis.

### 3.3 Pattern of intercellular communication between tumour and normal paracancerous tissues

**Figure 3:**
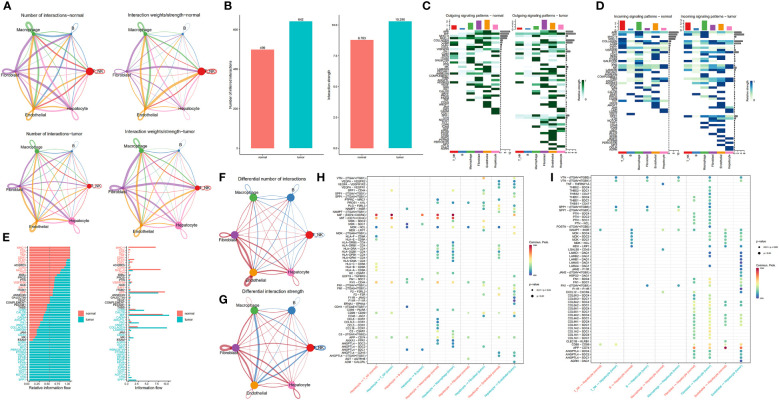
Comparison of cellular interactions between samples from tumour and adjacent normal tissues. **(A)** Cellular interaction number and strength. **(B)** Bar graph illustrating the total number (left) and weight (right) of ligand−receptor interactions between samples from tumour and adjacent normal tissues. **(C, D)** Heatmap showing possible afferent or efferent signalling pathways between cells. **(E)** Comparative profiles of pathway signal intensities indicating conserved and specific signalling pathways in tumour and normal tissue samples. **(F, G)** Communication quantity and intensity differences network. Red and blue colours represent upregulated and downregulated pathways, respectively, relative to normal tissues. **(H, I)** Dot plots show the variation in the signalling action of hepatocytes relative to other cell types.

We constructed a communication network between tumour samples and normal paracancerous samples to characterize alterations in signalling pathways (**Figure 3A**). A total of 642 and 499 significant ligand−receptor (LR) interactions were identified between the cell types present in tumour and normal paracancerous tissues, respectively (**Supplementary Table S2**). Differences between the number of communications among all cell populations between tumour and normal samples are illustrated in **Figure 3B**. In summary, tumour samples exhibited more cellular interactions than their normal counterparts, a phenomenon that was even more pronounced in the overall signalling pattern (Supplementary Figure S1). Next, we investigated the potential efferent and afferent signals among these six cell types and the specific molecular pairs. We found that the tumour samples consistently had more signal pairs than normal samples regardless of efferent or afferent signalling. The potential signalling pathways specific to tumour samples included SPP1, VTN, OCLN, CD46, GDF, EPHA, AGRN, PERIOSTIN, and HSPG. In normal samples, endothelial cells and T/NK cells were the main signalling providers and receptors, respectively, whereas in tumour samples, fibroblasts and macrophages represented the main signalling providers and receptors, respectively (**Figures 3C, D**). The overall communication probabilities of cells from tumour samples and normal sample sources were significantly different. Among the ligand receptors for intercellular communication in the normal sample sources, multiple pathways take part in inflammatory and immune responses, including pathways involving MHC-I, MHC-II, CXCL, complement, CCL, and TNF. In tumour samples, the intercellular interactions were mainly active in signalling pathways, including pathways involving SPP1, VTN, NOTCH, THY1, and CD46 (**Figure 3E**). To further elucidate the relationship between hepatocytes and other cell subpopulations, we generated a network plot of differences in number and strength. We found that hepatocytes had significantly higher interactions with endothelial cells and fibroblasts but a weaker association with immune cells (**Figure 3F-G**).

Differential analysis of all ligand−receptor pairs in hepatocytes and other cell types revealed significantly different patterns between tumour and adjacent normal tissues (**Figures 3H–I**). Studies have shown that CD74 promotes tumour cell growth by interacting with MIF (26). Remarkably, MIF-(CD74^+^CD44) signalling between hepatocytes and T/NK and macrophages, which mediates immunosuppressive effects that have previously been illustrated for promoting cancer progression (27). Blocking MIF-CD74 signalling not only inhibits the proliferation of HCC cells but also exerts antitumour effects. Therefore, MIF/CD74 axis inhibition could be an effective treatment for HCC (28). SPP1 encodes osteopontin (OPN), a phosphorylated glycoprotein expressed in various tissues and cells associated with human diseases (29, 30). Notably, OPN is crucial in tumour progression, including HCC metastasis and prognosis, since it drives the evolutionary adaptation of tumour cells in the tumour microenvironment. Strikingly, SPP1-CD44 signaling was present between hepatocytes and T/NK cells, macrophages, and fibroblasts in tumor samples, but not in normal samples adjacent to cancer, further supporting the critical role of SPP1 in the tumor ecosystem.

### 3.4 Transcriptome heterogeneity of hepatocytes in HCC

**Figure 4:**
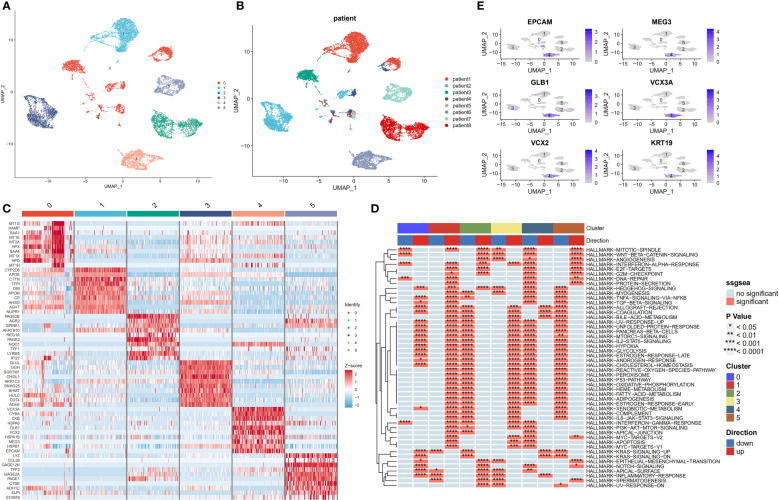
Transcriptome heterogeneity of malignant cells in HCC tissues. **(A)** UMAP plots of six different epithelial cell subpopulations. **(B)** UMAP plots demonstrating the heterogeneity among patients. **(C)** Heatmap of the top 10 differentially expressed genes(DEGs) across six epithelial cell clusters. **(D)** Single-cell pathway analysis of six subpopulations. **(E)** Scatter plot showing marker genes for cancer stem cells.

Despite previous batch effects, tumour cells continued to show patient-specific expression patterns. This suggests a high degree of heterogeneity, which could possibly be caused by CNVs. Six major cell subpopulations were identified after the entire malignant and normal hepatocytes reclustering (**Figure 4A**). In addition, UMAP plots revealed distinct clusters of malignant cells that corresponded to the sample origin (**Figure 4B**). **Figure 4C** illustrates the marker genes for each cell subpopulation. Next, the irGSEA package was employed to perform scRNA-seq gene set enrichment analysis and found that these subpopulations have unique activation signals. These signals include the Hedgehog signalling pathway (subpopulation 0), the early oestrogen response (subpopulation 1), the IL6/STAT3 and TNF signalling pathways (subpopulation 2), the xenobiotic metabolism and reactive oxygen species signalling pathways (subpopulation 3), and the KRAS signalling pathway (subpopulation 4). Moreover, multiple cell proliferation-related pathways were upregulated in subpopulation 5; these pathways included those involving the MYC targets V1 and V2, G2M checkpoints, E2F targets, WNT signalling, and P53 targets (**Figure 4D**). Activated KRAS is a major driver of cancer stem cell (CSC) proliferation and tumour metastasis (31). The results of the present study revealed that the KRAS signalling pathway was significantly upregulated in subpopulation 4, and the marker genes for CSCs were also distributed in this subpopulation (**Figure 4E**).

### 3.5 Profiles of chromosomal CNV in hepatocytes subpopulations

**Figure 5:**
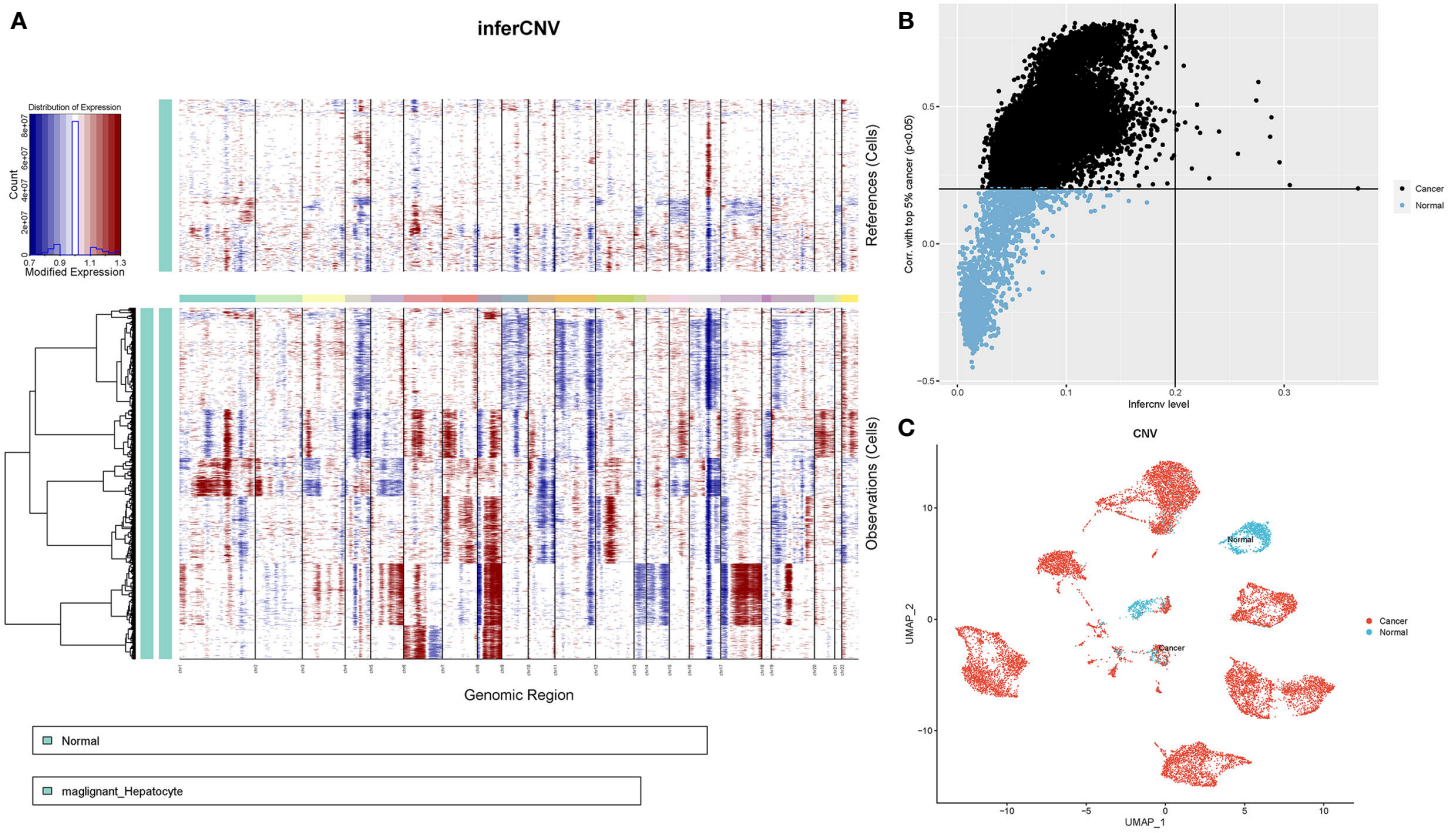
CNV analysis of HCC patient epithelial cells. **(A)** Heatmap showing CNV patterns in epithelial cells across 17 HCC samples. **(B)** Epithelial cells were classified as either malignant or nonmalignant. The horizontal coordinate represents the CNV value of the cell, whereas the vertical coordinate denotes the correlation coefficient of the top 5% of CNV values of tumour cells. **(C)** Distribution of tumour versus normal epithelial cells on the UMAP plot based on copy number variation.

Next, we determined the chromosomal CNVs in each sample based on transcriptomic data to understand the malignancy of the epithelial cell subpopulation. This image shows the results revealed low and high CNV in adjacent normal epithelial cell subpopulations (control samples) and tumour epithelial cells, respectively. Chromosome amplification primarily occurs within chromosomes 1, 3, 5, 6, 7, 8, 12, 15, 17, 20, 21, and 22, with deletions most commonly observed in chromosomes 4, 9, 10, 11, 13, 14, 16 and 18 (**Figure 5A**). First, the copy value (CNV value) was calculated based on the sum of squares for all genes in each sample. Next, we ranked the CNV values of the tumour cells, using the top 5% as a reference, and then calculated correlation coefficients between other epithelial and reference cells. The determination of tumour cells was achieved at a threshold CNV >0.2 and a correlation coefficient >0.2. With CNV value as the horizontal coordinate and correlation coefficient as the vertical coordinate, black dots represent tumor cells and blue dots represent normal cells (**Figure 5B**). Finally, 13,502 tumor cells and 1,718 normal cells were identified and projected on the UMAP map (**Figure 5C**).

Thereafter, we employed the FindAllMarkers function and set the screening conditions logfc = 0.25 (difference multiplicity), min. pct = 0.25 (minimum differential gene expression ratio) and pct. diff >0.1 (pct.1-pct.2) to identify marker genes in the hepatic malignant and normal epithelial cell subsets. The results revealed a total of 564 marker genes (**Supplementary Table S3**). We hypothesize that their function in HCC differs from that in normal epithelial cells, although further research exploration is needed.

### 3.6 Malignant hepatocyte subpopulations are associated with HCC prognosis

**Figure 6:**
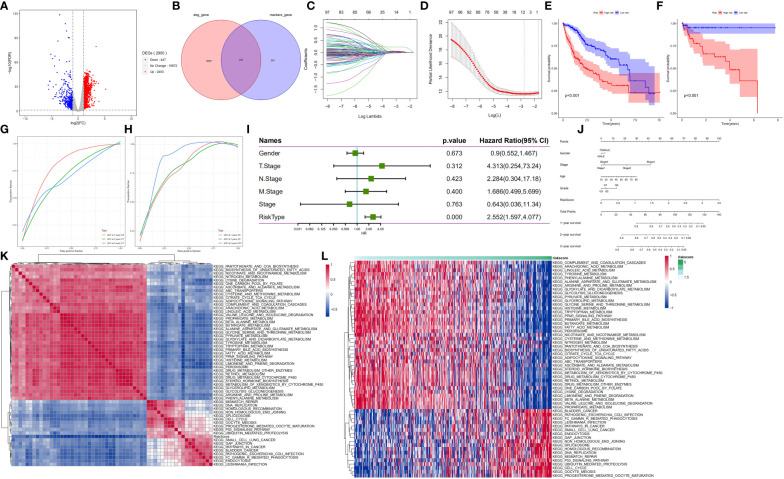
HDG identification and validation in the training (TCGA-LIHC) and validation cohorts (GSE76427). **(A)** The volcano plot of DEGs in the TCGA-LIHC dataset. **(B)** The intersection of DEGs of TCGA-LIHC cohort with marker genes of epithelial cell subpopulation of HCC. **(C, D)** Coefficient distribution plots of log(λ) sequences **(C)** and selection of optimal parameters (lambda) in the LASSO model **(D)**. **(E, F)** Kaplan−Meier survival curves illustrate the prognostic value of the 11-gene signature in the training cohort **(E)** and validation cohort **(F)**. **(G, H)** Distribution of the 11-gene signature risk scores and survival status of HCC patients in the training cohort **(G)** and validation cohort **(H)**. ROC curves showing the value of the 11-gene signature in predicting the OS rates of HCC patients at 1, 3, and 5 years in both cohorts. **(I)** Forest plot showing multivariate Cox analysis results. **(J)** Nomogram showing the prediction of OS at 1, 2, and 3 years. **(K, L)** Regulatory pathways potentially related to risk score.

Next, we explored the prognostic role of hepatocyte subpopulations in HCC patients. Analysis of the mRNA expression data from HCC samples across the TCGA database yielded 2,900 differentially expressed genes (**Figure 6A**). Marker genes from malignant and nonmalignant cells of hepatocyte subpopulations intersected with DEGs related to HCC development in the TCGA database. Notably, 2,900 DEGs overlapped with 564 marker genes, resulting in 203 differentially expressed marker genes in HCC. These were subsequently named hepatocyte differential genes (HDGs) (**Figure 6B**). Univariate Cox regression analysis revealed 101 differentially expressed marker genes that were significantly related to the prognosis of HCC patients. To obtain a more robust prognostic profile, we employed the LASSO regression algorithm at 10-fold cross-validation with a lambda-min of 0.06321515 to designate a prognostic model consisting of 11 genes, namely, MARCKSL1(MARCKS Like 1), SPP1 (Secreted Phosphoprotein 1), BSG(Basigin, also called CD147 or EMMPRIN), CCT3 (chaperonin containing TCP1 subunit 3), LAGE3 (L antigen family member 3), KPNA2 (karyopherin subunit alpha 2), SF3B4 (Splicing Factor 3b Subunit 4), GTPBP4 (GTP Binding Protein 4), PON1 (Paraoxonase 1), CFHR3 (Complement factor H-related 3) and CYP2C9(cytochrome P450 family 2 subfamily C member 9) (**Figures 6C, D**).

Next, the median risk score was used to stratify the patients into high- and low-risk groups. Patients in the low-risk group showed significantly higher OS rates than their counterparts in the high-risk group (*p<0.001*) (**Figure 6E**). Application of the 11-gene signature in the validation cohort also indicated that patients in the low-risk group had longer OS rates than their counterparts in the high-risk group (*p<0.001*) (**Figure 6F**). To test the prognostic performance of the 11-gene signature, time-dependent ROC curves were generated targeting TCGA-LIHC samples. The results revealed area under the curve (AUC) values of 0.8, 0.7, and 0.7 for 1-, 3- and 5-year survival, respectively, in the testing cohort (**Figure 6G**) and 0.8, 0.8, and 0.87, respectively, in the validation cohort (GSE76427) (**Figure 6H**). These findings suggest that the 11-gene signature had good prognostic value in both cohorts. For the association analysis between the clinicopathological characteristics and the prognostic model, we analysed gender, TNM, stage, and risk scores in the TCGA-LIHC sample. The multivariate Cox regression analysis results revealed that the risk score was a significant independent prognostic factor for patients with LIHC (*p<0.001*) (**Figure 6I**). Moreover, we generated a nomogram encompassing gender, stage, age, grade, risk score and 1-, 2- and 3-year survival. Next, we employed a one-sample GSEA approach to calculate scores for each sample across 175 pathways based on the risk score to identify relevant regulatory pathways. Thereafter, the correlation between each pathway and the risk score was no less than 0.3 for the evaluation. The results revealed 39 positive and 50 negative correlations with the sample risk score. Pathways that were positively correlated with the risk score included those related to cancer development, whereas the negatively correlated pathways included those regulating glycolysis/glycogenesis, glycine, and the metabolism of fatty acids, serine, threonine, glyoxylate and dicarboxylate (**Figures 6K, L**).

### 3.7 The relative RNA expression level and protein expression level of MARCKSL1, SPP1, BSG, CCT3, LAGE3, KPNA2, SF3B4, GTPBP4, PON1, CFHR3 and CYP2C9

Based on the initial trend of differentially up- and down-regulated genes (**Supplementary Table S4**), To further investigate the gene expression characteristics of 11 prognosis-related differentially expressed genes (MARCKSL1, SPP1, BSG, CCT3, LAGE3, KPNA2, SF3B4, GTPBP4, PON1, CFHR3 and CYP2C9) in the high-risk and low-risk groups of HCC patients, we performed a correlation analysis between gene expression levels and risk scores. The results showed that all eight genes were positively correlated with risk scores, except for PON1, CFHR3 and CYP2C9, whose mRNA expression levels were significantly negatively correlated with risk scores (**Supplementary Figure S3A**). Meanwhile, in order to classify the high and low risk genes, we could see from the forest plot of 11 prognostic genes that the hazard ratio of MARCKSL1, SPP1, BSG, CCT3, LAGE3, KPNA2, SF3B4 and GTPBP4 were all greater than 1, suggesting that these 8 genes might be poor prognostic factors and belong to high risk genes, while PON1, CFHR3 and CYP2C9 were all less than 1, suggesting that these three genes may be factors with a better prognosis (**Supplementary Figure S3B**).

**Figure 7:**
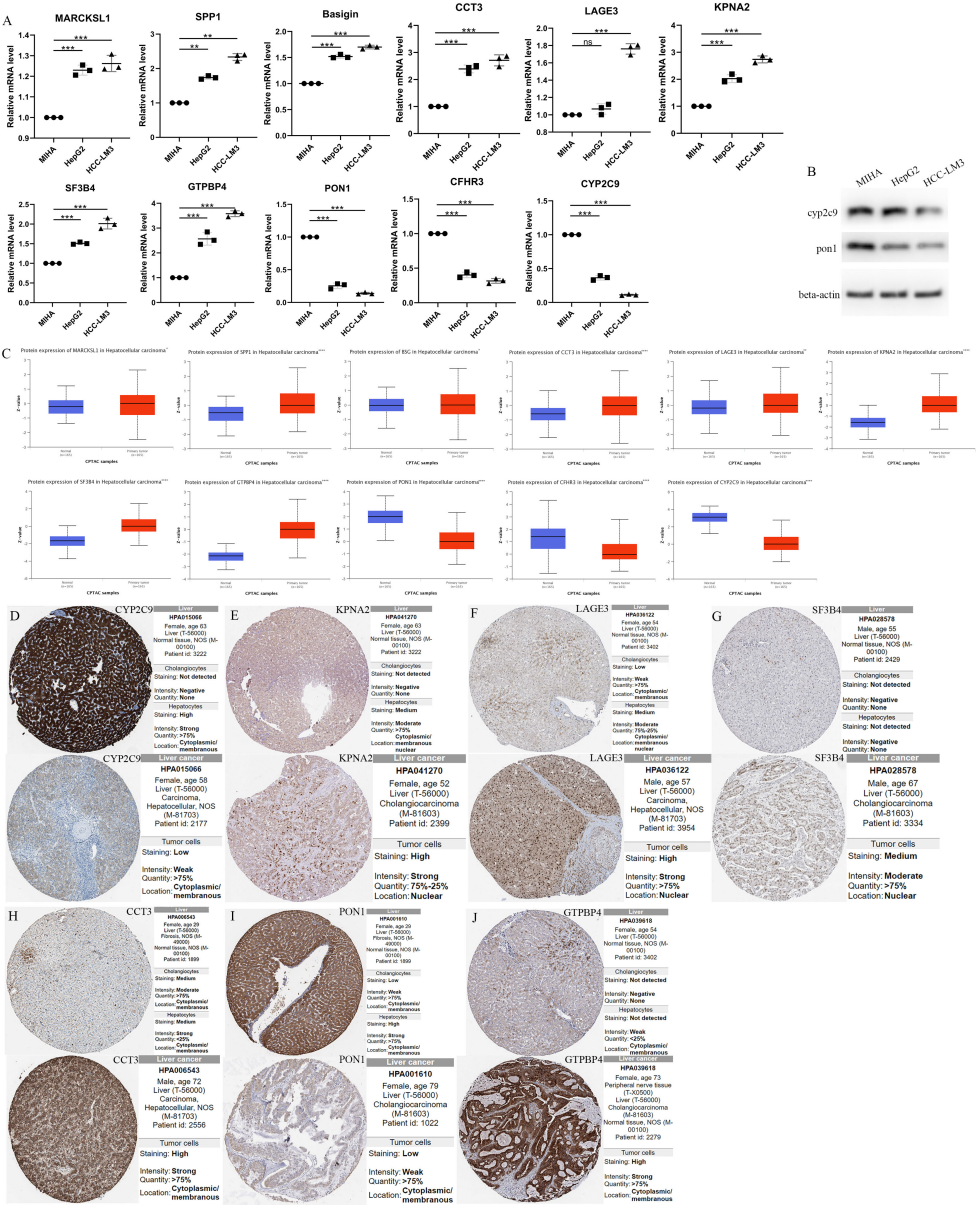
The Relative RNA Expression Level and Protein Expression Level of prognosis-related differentially expressed genes. **(A)** The Relative RNA Expression Level of MARCKSL1, SPP1, BSG, CCT3, LAGE3, KPNA2, SF3B4, GTPBP4, PON1, CFHR3 and CYP2C9. **(B)** Expression of CYP2C9 and PON1 in normal human hepatocyte cell line MIHA and HCC cell lines HCC-LM3 and HepG2 through western blot analysis. **(C)** Box plots showed the differential protein expression of 11 hub genes in the CPTAC dataset in HCC tumor tissue and adjacent normal. **(D-J)** Immunohistochemical analysis of the CYP2C9, KPNA2, LAGE3, SF3B4, CCT3, PON1 and GTPBP4 in HCC and liver tissues from the HPA database. HCC, hepatocellular carcinoma; CPTAC, The National Cancer Institute’s Clinical Proteomic Tumor Analysis Consortium. HPA, Human Protein Atlas. (Unpaired t-test, **P < 0.01, ***P < 0.001, ****p < 0.0001 and ns, no significance).

The RNA expression of MARCKSL1, SPP1, BSG, CCT3, LAGE3, KPNA2, SF3B4, GTPBP4, PON1, CFHR3 and CYP2C9 in normal human hepatocyte cell line MIHA and HCC cell lines HCC-LM3(high metastatic HCC cells) and HepG2(low metastatic HCC cells) were compared by qPCR. It was found that CYP2C9, PON1 and CFHR3 were low expressed and MARCKSL1, SPP1, BSG, CCT3, LAGE3, KPNA2, SF3B4, GTPBP4 were over expressed in human hepatoma cells compared with normal human hepatocyte cells (Unpaired t-test, p<0.01) (**Figure 7A**). **Figure 7B** shows the results of the protein expression levels of CYP2C9 and PON1 were down regulated in HepG2 and HCC-LM3 compared to MIHA. At the same time, CPTAC database analysis results showed that the protein expression of PON1, CFHR3 and CYP2C9 were low expressed in tumor tissues compared with paracancer normal tissue, while other genes were over expressed (**Figure 7C**).

Furthermore, immunohistochemical analysis from HPA database confirmed higher KPNA2, LAGE3, SF3B4, CCT3 and GTPBP4 protein expression and lower CYP2C9 and PON1 protein expression in HCC tissues (**Figures 7D–J**).

### 3.8 Drug sensitivity analysis of risk models and targeting of potential compounds in high risk groups using connectivity map (CMap)

**Figure 8:**
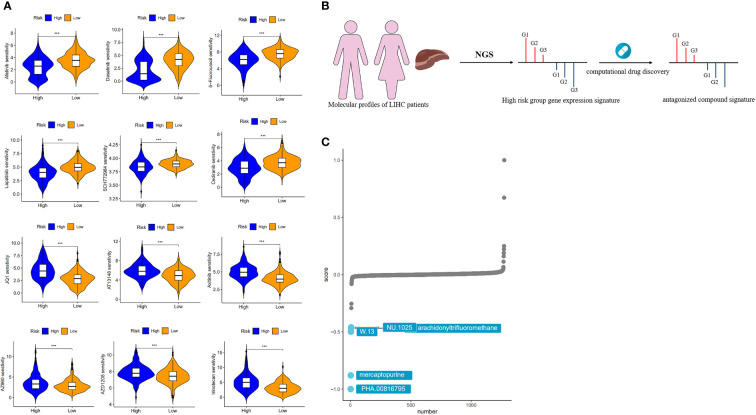
Drug sensitivity analysis and target compound screening for risk models. **(A)** Sensitivity analysis of chemotherapeutic agents between different risk groups. **(B)** How the “signature reversion”-based calculation method works. **(C)** The top 5 drugs with the lowest CMap scores.

To determine the impact of risks on clinical practice, we evaluated the IC50 values of several chemotherapeutic agents in the high- and low-risk groups using the “oncoPredict” package. This analysis identified 123 drugs that were statistically significant (*p < 0.01*) (**Supplementary Table S5**). The results showed that afatinib, dasatinib, 5-fluorouracil, lapatinib, SCH772984, and cediranib had lower IC50 values in the high-risk group than in the low-risk group, suggesting that patients in the high-risk group may benefit more from these drugs. In contrast, JQ1, AT13148, axitinib, AZ960, AZD1208, and irinotecan had lower IC50 values in the low-risk group, suggesting that low-risk patients may benefit more from the above chemotherapeutic agents (**Figure 8A**).

While single-cell sequencing strategies are powerful tools for constructing disease signatures specific to individual cell types, CMap provides unprecedented convenience for researchers to tightly link the triad of drug, gene and disease in a context where deep understanding is lacking, as this method does not require the detailed mechanism of action or drug target to be provided in advance to predict therapeutic potential. Therefore, by combining a high-resolution single-cell sequencing strategy with CMap, we have been able to directly target effective therapeutic agents based on individual cell-level expression signatures and thus provide a more accurate prediction for screening potential drugs for disease.

We used a computational drug discovery strategy based on “signature reversion” (32) to identify drugs with a high risk of reversion using the large amount of data in the CMap database (**Figure 8B**). The top 300 genes with the highest fold change in the high- and low-risk groups were extracted for XSum analysis (**Supplementary Table S6**). The results of the CMap analysis revealed several compounds with gene expression patterns opposite to those specific to the high-risk group, with lower CMap scores indicating a higher perturbation ability. PHA.00816795, mercaptopurine, W.13, NU.1025 and arachidonyltrifluoromethane were the five potentially valuable small molecule drug candidates, as they were ranked as the top 5 candidates (**Figure 8C**). Among the top three of these candidates is mercaptopurine, which is a common chemotherapeutic drug that produces anticancer effects by interfering with cell division or DNA synthesis (33). Yu et al. obtained five drugs associated with HCC by integrating multiple data to define the types of genes, considering the effect of genetic changes on HCC and the positive and negative relationships between drugs and HCC (34). Among these drugs, mercaptopurine is a potential anti-HCC drug.

## 4 Discussion

The liver is a major site for many metabolic processes, and metabolic dysregulation is vital for HCC progression and development (35). Evidence from numerous studies has shown that HCC originates from adult hepatocytes (36, 37). In this study, we found that HCC occurs in adult hepatocytes. Moreover, there were metabolic changes in the hepatic epithelial cells. While normal hepatocytes produce energy primarily through oxidative phosphorylation, malignant hepatocytes convert glucose into lactate through glycolysis to generate energy, a phenomenon known as the Warburg effect (38). The dysregulation of oxidative phosphorylation is related to elevated HCC tumorigenicity (39, 40). The liver synthesizes lactic acid and can store and breakdown lipids. Therefore, in HCC, aberrant lipid metabolism generates the lipids required for membrane formation and energy production, and posttranslational modifications support tumorigenesis (41). In our study, the gluconeogenic pathway (aerobic gluconeogenesis) was found to be enhanced in normal hepatic epithelial cells adjacent to cancerous cells, whereas the lipid metabolism pathway was enriched in malignant hepatocytes (**Figure 2E**).

HCC is a heterogeneous disease influenced by multiple factors, which makes it difficult to diagnose and perform individualized treatment. HCC patients are often diagnosed after curative surgical approaches are no longer possible because these patients are at an advanced stage of the disease. Traditional sequencing methods often mask the underlying heterogeneity in phenotypically defined cell subpopulations. In contrast, scRNA-seq allows the in-depth exploration of tumour heterogeneity and the analysis of tumour development, drug resistance, intercellular communication and immune infiltration patterns (12). Thus, this technique was employed to comprehensively analyse the HCC landscape at single-cell resolution.

To understand the interactions among hepatocytes and stromal cells and immune cells, we conducted intercellular communication analysis. This revealed enhanced interactions between hepatic epithelial cells and fibroblasts and reduced contact with immune cells, macrophages and endothelial cells in tumour samples compared to normal adjacent samples (Supplementary Figure S2). Cancer-associated fibroblasts (CAFs) are a major part of the tumour stroma and contribute to HCC progression. Furthermore, CAFs interact with tumour cells, immune cells, or vascular endothelial cells in the TME through direct intercellular contacts or indirect paracrine interactions to promote HCC (42–44). Similarly, Wang et al. performed a single-cell level analysis of samples from normal and malignant livers and found that in HCC, the most significant alteration was the expansion of ACTA2+ fibroblast populations and malignant cells. This suggests that the transition of hepatocytes from normal to malignant is accompanied by alterations in intercellular contact with other cells in the tumour microenvironment, which produce the complex intra- and intertumoral heterogeneity of HCC (45).

Differentially expressed genes between malignant and normal hepatocytes were identified by analysing copy number variations in single-cell transcriptome data and isolating malignant and nonmalignant cells from hepatocytes. In addition, analysis of TCGA-LIHC survival data revealed the molecular markers associated with HCC prognosis,including MARCKSL1, SPP1, BSG, CCT3, LAGE3, KPNA2, SF3B4, GTPBP4, PON1, CFHR3, and CYP2C9. The identified prognostic risk factors showed good prediction performance in both HCC cohorts. Based on this, we also constructed a nomogram risk assessment model, which combines risk scores with clinical characteristics to facilitate the clinical application of HCC. It has been reported that CYP2C9 is involved in the metabolism of many carcinogens and drugs, and is down-regulated in HCC (46). Wang et al. used time serial transcriptome to reveal that *Cyp2c29* is a key gene in the development of hepatocellular carcinoma in the mouse model, and its overexpression enhances the production of 14,15-EET and inhibits inflammation induced hepatocellular proliferation by inhibiting the IKK-NF-κB pathway during liver injury (47). Meanwhile, the expression of the human homologous of *Cyp2c29* gene in mice was positively correlated with the survival time of HCC patients, further suggesting that CYP2C epoxygenases may be a potential therapeutic target for liver disease. Chen and others have revealed lncZic2/depletion/MARCKS/MARCKSL1 pathways can eliminate the liver tumor–initiating cells (TICs) (48). The overexpression of myristoylated alanine-rich protein kinase C substrate (MARCKS) and MARCKS like 1(MARCKSL1) can drive the self-renewal of TICs. Yang et al. demonstrated that BSG may be a tumor-promoting factor in HCC (49). The potential diagnostic role of BSG in differentiating HCC specimens from non-tumor specimens was demonstrated by analysis of multiple cohorts. BSG mRNA expression levels were significantly upregulated in both HCC specimens and HCC cell lines, and significantly shorter Overall Survival (OS) (*P* = 0.0014) and Disease Free Survival (DFS) (*P* = 0.0097) were observed in patients with high BSG expression relative to those with low BSG expression. Han et al. revealed that CCT3 is a new complementary biomarker for HCC screening and diagnosis (50). Several studies have shown that CCT3 is overexpressed in HCC patients by quantitative RT-PCR and western blotting. CCT3 can influence the progression of HCC by affecting phosphorylation signaling and translocation of STAT3/STAT3 into the nucleus of HCC cells (51, 52). The study of Li et al. showed that LAGE3 has prognostic value in HCC, which may affect the progression path of HCC tumor by promoting the proliferation, survival, migration, invasion and anti-apoptosis of HCC cells through the PI3K/AKT/mTOR and Ras/RAF/MAPK pathways (53). Guo et al. identified KPNA2 as a potential diagnostic and prognostic biomarker for HCC, which may affect HCC cell proliferation and migration by regulating cell cycle and DNA replication (54). Splicing factor 3b subunit 4 (SF3B4) has been revealed to be associated with the diagnosis and prognosis of HCC (55, 56). Liu et al. further demonstrated that SF3B4 drives cell proliferation and metastasis in HCC (57). Deng et al. further studied the mechanism and revealed the interaction between SF3B4 and ENAH in HCC, that is, SF3B4-regulated ENAH promotes the development of HCC by activating Notch signaling (58). It has been reported that Guanosine triphosphate binding protein 4 (GTPBP4) is associated with poor prognosis in HCC patients (59). Additional reports have explored the role of GTPBP4 in metabolic regulation and the potential mechanisms involved in HCC development and metastasis (60). GTPBP4 induces the dimer conformation of PKM2 through the SUMOylation to promote the aerobic glycolysis of HCC, thus promoting the progression and metastasis of HCC (61). Serum Paraoxonase 1 (PON1) has been reported as a biomarker for evaluating microvascular infiltration in hepatocellular carcinoma. Complement factor H related 3 (CFHR3) can be used to predict the prognosis of HCC. Overexpression of CFHR3 can affect the proliferation and apoptosis of hepatocellular carcinoma (62). Recent reports suggest that overexpression of CFHR3 may be a potential strategy for overcoming hypoxia and treating HCC (63). These studies confirm the significance and plausibility of these prognostic signatures.

Currently, liver transplantation and resection are efficient treatment options for early-stage disease; however, these treatments are appropriate for only 20-30% of HCC patients (64). Chemotherapy is another viable treatment option for advanced HCC. Recently, there has been significant progress in the development of molecularly targeted treatments for liver cancer (65). These include sorafenib, levatinib, and regorafenib, which have been approved as first- and second-line treatments for HCC. In this study, the sensitivities of HCC to various treatments were predicted. Low-risk patients showed higher sensitivity to afatinib, dasatinib, 5-fluorouracil, lapatinib, SCH772984, and cediranib than high-risk patients, which may be attributed to their higher metabolic activity. Various drugs were suggested for low-risk group patients, such as JQ1, AT13148, axitinib, AZ960, AZD1208, and irinotecan. Cancerous cells have the potential to evade the immune system (66). Immune escape can be achieved through a variety of mechanisms. Thus, therapeutic strategies that block checkpoint inhibitors of the PD-1/PD-L1 and CTLA-4 pathways can promote tumour-reactive T-cell aggregation, thereby improving the antitumour response (67, 68).

To the best of our knowledge, the 11-gene signature is the first to explore the overall molecular prognostic feature of subpopulations associated with metabolic disorders from single-cell sequencing data. This risk model exhibited excellent ability to predict the prognosis of HCC patients, and the AUC values at year 1, year 3 and year 5 were all greater than 0.7, with the optimal value of 0.8. Meanwhile, a novel XSum algorithm was used to predict potential drugs targeting high-risk groups from the Cmap database, and 5 drugs were finally obtained, including PHA.00816795, mercaptopurine, W.13, NU. 1025 and arachidonyl trifluoromethane. Small molecule drugs, serving as candidates, embrace potential value conducive to providing medication strategies for accurate treatment of HCC patients.

This research also has certain drawbacks. First, more perspective data with larger sample size should be collected to validate the accuracy of our established prognostic model. Second, the characteristics of different fractionated epithelial cells have not been generated and validated. Further in-depth analysis from specific epithelial cell subtypes closely related to metabolic changes will be conducive to obtaining more accurate and valid prognostic characteristics. Nevertheless, scRNA-seq analysis sheds new light on the metabolic characteristics of individual cell subsets in HCC, and anchors the survival and prognosis of relevant cell subsets with the most significant metabolic changes, which is a key step forward in clinical practice.

In conclusion, the present study identified prognostic genes significantly associated with metabolic changes in a hepatocyte subpopulation at the single-cell level, and explored the heterogeneity of this subpopulation and its interrelationships with other cells in the tumor microenvironment. A prognostic model for OS prediction in HCC patients was established and validated and the results demonstrated favourable predictive ability. Additionally, differences in chemosensitivity between high-risk and low-risk groups were evaluated, and five potential drugs that might reverse the risk score were forecasted. These results provided an in-depth understanding of the metabolic characteristics of HCC. Furthermore, the characteristics of potential prognostic biomarker can be clarified through the comparison of tumor-related genes constructed by liver malignant cells and normal hepatocytes. The above may be conducive to new strategies of individualized therapy.

## Data availability statement

The original contributions presented in the study are included in the article/**Supplementary Material**. Further inquiries can be directed to the corresponding authors.

## Ethics statement

Ethical approval was not provided for this study on human participants because TCGA and GEO belong to public databases. The patients involved in the database have obtained ethical approval. Users can download relevant data for free for research and publish relevai1t articles. Our study is based on open source data, so there are no ethical issues and other conflicts of interest. The Cancer Genome Atlas (TCGA), a landmark cancer genomics program, molecularly characterized over 20,000 primary cancer and matched normal samples spanning 33 cancer types. This joint effort between NCI and the National Human Genome Research Institt1te began in 2006, bringing together researchers from diverse disciplines and multiple institt1tions. Over the next dozen years, TCGA generated over 2.5 petabytes of genomic, epigenomic, transcriptomic, and proteomic data. The data, which has already led to improvements in our ability to diagnose, treat, and prevent cancer, will remain publicly available for anyone in the research community to use. (https://www.cancer.gov/about-nci/organization/ccg/research/structural-genomics/tcga). Written informed consent for participation was not required for this study in accordance with the national legislation and the institutional requirements.

## Author contributions

CH and JC contributed conception and design of the study; ZH and HY refined the research design idea. JC and JH collected the data; CH and JC performed the statistical analysis; JH and RZ accomplished the RT-qPCR assay and Western blot; CH and JZ further supplemented and validated the data and assisted with the interpretation of the results; CH and JC drafted the manuscript; ZH and HY revised the logic of the manuscript and polished the language. All authors contributed to manuscript and approved the submitted version.
