# Corrigendum: Single-cell transcriptome analysis reveals the metabolic changes and the prognostic value of malignant hepatocyte subpopulations and predict new therapeutic agents for hepatocellular carcinoma

**DOI:** 10.3389/fonc.2023.1162375

**Published:** 2023-03-08

**Authors:** Cuifang Han, Jiaru Chen, Jing Huang, Riting Zhu, Jincheng Zeng, Hongbing Yu, Zhiwei He

**Affiliations:** ^1^ Guangdong Provincial Key Laboratory of Medical Molecular Diagnostics, The First Dongguan Affiliated Hospital, Guangdong Medical University, Dongguan, China; ^2^ School of Pharmacy, Guangdong Medical University, Dongguan, China; ^3^ Dongguan Key Laboratory of Medical Bioactive Molecular Developmental and Translational Research, Guangdong Provincial Key Laboratory of Medical Molecular Diagnostics, Guangdong Medical University, Dongguan, China

**Keywords:** cancer metabolism, hepatocellular carcinoma, malignant hepatocytes, prognostic biomarker, single-cell RNA sequencing

In the published article, there was an error in **Figure 6** as published. The image of **Figure 6G** is superimposed on **Figure 6H**. The corrected **Figure 6** and its caption **Figure 6** HDG identification and validation in the training (TCGA-LIHC) and validation cohorts (GSE76427).

**Figure 6:**
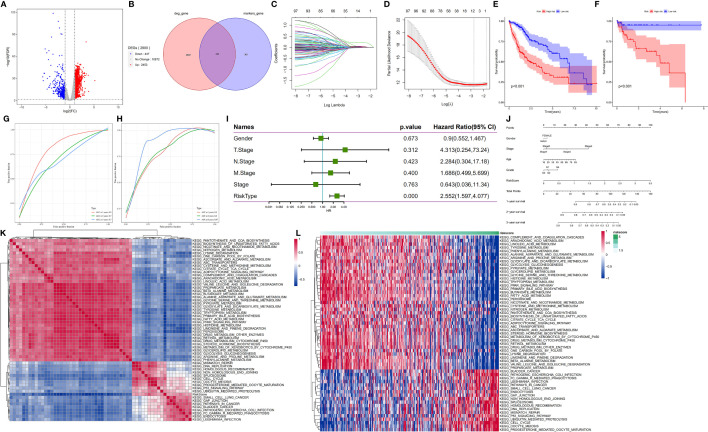
HDG identification and validation in the training (TCGA-LIHC) and validation cohorts (GSE76427). **(A)** The volcano plot of degs in the TCGA-LIHC dataset. **(B)** The intersection of degs of TCGA-LIHC cohort with marker genes of epithelial cell subpopulation of HCC. **(C, D)** Coefficient distribution plots of log(λ) sequences **(C)** and selection of optimal parameters (lambda) in the LASSO model **(D)**. **(E, F)** Kaplan−Meier survival curves illustrate the prognostic value of the 11-gene signature in the training cohort **(E)** and validation cohort **(F)**. **(G, H)** Distribution of the 11-gene signature risk scores and survival status of HCC patients in the training cohort **(G)** and validation cohort **(H)**. ROC curves showing the value of the 11-gene signature in predicting the OS rates of HCC patients at 1, 3, and 5 years in both cohorts. **(I)** Forest plot showing multivariate Cox analysis results. **(J)** Nomogram showing the prediction of OS at 1, 2, and 3 years. **(K, L)** Regulatory pathways potentially related to risk score.

In the published article, there was an error in **FIGURE 7** as published. The symbols used for the statistical significance analysis in **Figure 7A** and **Figure 7C** are superimposed. The corrected **Figure 7** and its caption **Figure 7** The Relative RNA Expression Level and Protein Expression Level of prognosis-related differentially expressed genes. Appear below.

**Figure 7:**
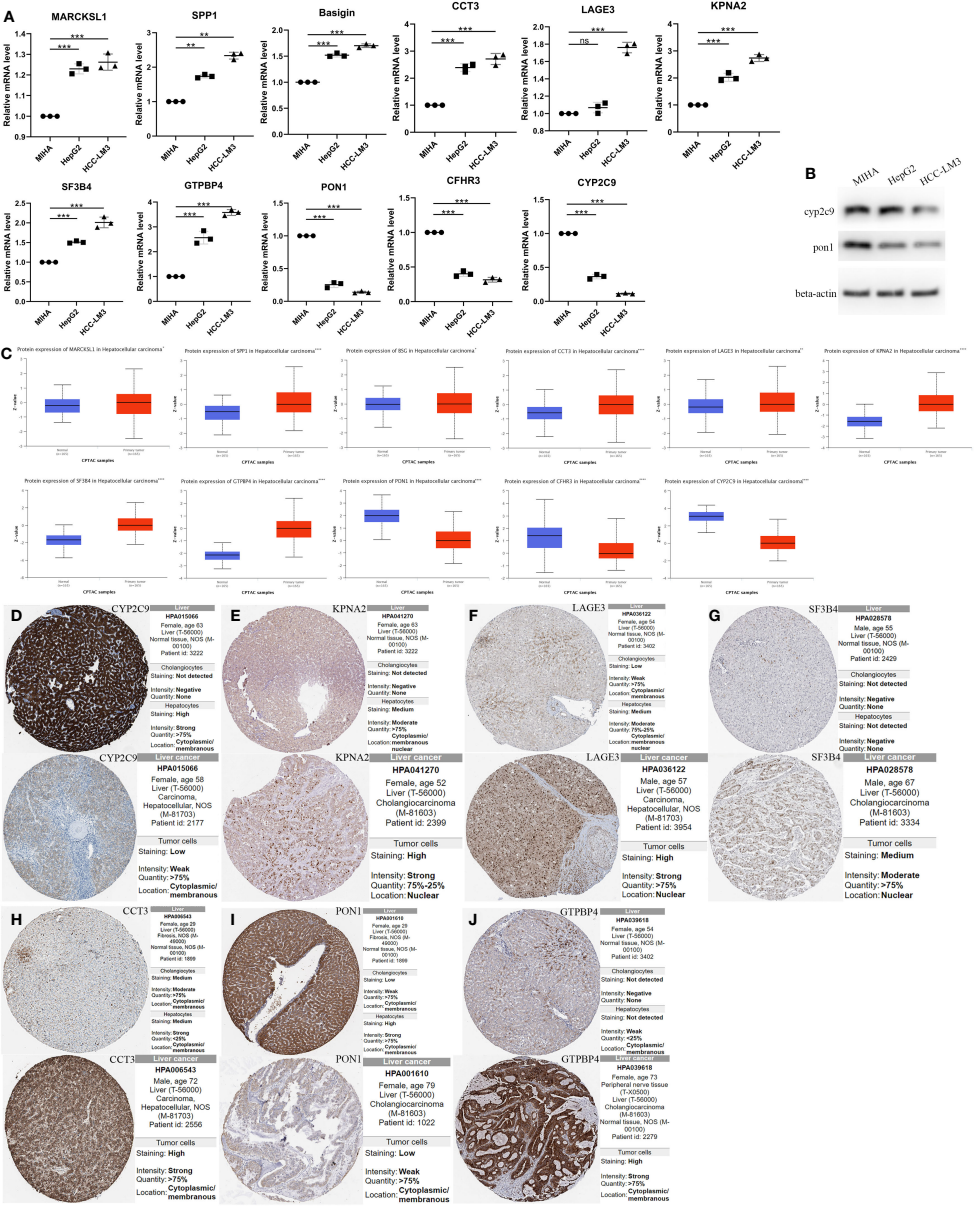
The Relative RNA Expression Level and Protein Expression Level of prognosis-related differentially expressed genes. **(A)** The Relative RNA Expression Level of MARCKSL1, SPP1, BSG, CCT3, LAGE3, KPNA2, SF3B4, GTPBP4, PON1, CFHR3 and CYP2C9. **(B)** Expression of CYP2C9 and PON1 in normal human hepatocyte cell line MIHA and HCC cell lines HCC-LM3 and HepG2 through western blot analysis. **(C)** Box plots showed the differential protein expression of 11 hub genes in the CPTAC dataset in HCC tumor tissue and adjacent normal. **(D–J)** Immunohistochemical analysis of the CYP2C9, KPNA2, LAGE3, SF3B4, CCT3, PON1 and GTPBP4 in HCC and liver tissues from the HPA database. HCC, hepatocellular carcinoma; CPTAC, The National Cancer Institute’s Clinical Proteomic Tumor Analysis Consortium. HPA, Human Protein Atlas. (Unpaired t-test, *P < 0.05, **P < 0.01, ***P < 0.001, ****p < 0.0001 and ns, no significance).

The authors apologize for these errors and state that they do not change the scientific conclusions of the article in any way. The original article has been updated.

