# Supplementary material for: Single-cell transcriptome analysis reveals the metabolic changes and the prognostic value of malignant hepatocyte subpopulations and predict new therapeutic agents for hepatocellular carcinoma
Source: Front Oncol. 2023 Jan 31;13:1104262. doi: 10.3389/fonc.2023.1104262 (PMC9969971; doi:10.3389/fonc.2023.1104262)
Supplement: Supplementary file 1 [file DataSheet_1.zip › Supplementary Material/Figure and table captions.DOCX]

Supplementary Figure S1: Heatmap depicting signals contributing the most to the overall signaling pathways in both paracancer normal samples and hepatocellular carcinoma samples.

Supplementary Figure S2: Circos plots displaying putative ligand-receptor interactions between hepatocytes and other cell clusters from normal group (left) and tumor (right) group. The brand links pairs of interacting cell types, and corresponding number of events were labeled in the graph.

Supplementary Figure S3: (A) Correlation between prognostic genes and risk scores. (B) Forest map showing the 11-gene signature identified by multivariate Cox regression analysis of OS.

Supplementary Table S1: Primer sequences of MARCKSL1, SPP1, BSG, CCT3, LAGE3, KPNA2, SF3B4, GTPBP4, PON1, CFHR3 and CYP2C9 genes and internal reference gene beta Actin (ACTB).

Supplementary Table S2: A communication network between tumour samples and normal paracancerous samples to characterize alterations in signalling pathways were constructed. A total of 642 and 499 significant ligand‒receptor (LR) interactions were identified between the cell types present in tumour and normal paracancerous tissues, respectively.

Supplementary Table S3: A total of 564 marker genes were identified using the FindAllMarkers function and set the screening conditions logfc = 0.25 (difference multiplicity), min. pct = 0.25 (minimum differential gene expression ratio) and pct. diff >0.1 (pct.1-pct.2) in the hepatic malignant and normal epithelial cell subsets.

Supplementary Table S4: A correlation analysis between gene expression levels and risk scores of 11 prognosis-related differentially expressed genes (MARCKSL1, SPP1, BSG, CCT3, LAGE3, KPNA2, SF3B4, GTPBP4, PON1, CFHR3 and CYP2C9) in the high-risk and low-risk groups of HCC patients.

Supplementary Table S5: Drug sensitivity analysis of risk models and targeting of potential compounds in high risk groups using Connectivity Map (CMap). Using the "oncoPredict" package to evaluate the IC50 values of several chemotherapeutic agents in the high- and low-risk groups and identified 123 drugs that were statistically significant (p < 0.01).

Supplementary Table S6: We used a computational drug discovery strategy based on "signature reversion" to identify drugs with a high risk of reversion using the large amount of data in the CMap database. The top 300 genes with the highest fold change in the high- and low-risk groups were extracted for XSum analysis.
