# Supplementary material for: Single-cell transcriptome analysis reveals the metabolic changes and the prognostic value of malignant hepatocyte subpopulations and predict new therapeutic agents for hepatocellular carcinoma
Source: Front Oncol. 2023 Jan 31;13:1104262. doi: 10.3389/fonc.2023.1104262 (PMC9969971; doi:10.3389/fonc.2023.1104262)
Supplement: Supplementary file 1 [file DataSheet_1.zip › Supplementary Material/Table S1.DOCX]

Table S1. Primer sequences of HCC prognostic genes and internal reference gene beta Actin (ACTB)

| Primer name | Sense | Anti-sense |
| --- | --- | --- |
| MARCKSL1  KPNA2 | CTCCATTGACTCGAACGACTC  CTGTTGGCTCTCCTTGCAGTTC | GCAGCTTAGAGATCACCCACCT GCAGGATTCTTGTTGCGGCAAAG |
| GTPBP4 | CCGTTTGCCAACCATTGATCCG | GTTGTGAACGCATAGGGCTGGA |
| CYP2C9 | CAGAGACGACAAGCACAACCCT | ATGTGGCTCCTGTCTTGCATGC |
| CFHR3 | AGCAGATGGAAATTCTTCAGGATC | AGTAGAAAGGAGGTGGTATCACC |
| CCT3 | GGAGGAAGAGTACATCCAGCAG | CCGCATAAGGTAGTGCTGAGCT |
| Basigin | GGCTGTGAAGTCGTCAGAACAC | ACCTGCTCTCGGAGCCGTTCA |
| LAGE3 | CAAAGGGTGGTTGGGAAGGATC | AGTTGATGACGGAAATTCGGAGC |
| SF3B4 | GGATGAGAAGGTTAGTGAACCGC | GGCATAGTCAGCATCTTCCTCAC |
| SPP1 | CGAGGTGATAGTGTGGTTTATGG | GCACCATTCAACTCCTCGCTTTC |
| PON1 | ATGCTCTCCGAGAGGTACAACC | GCCAGTCCATTAGGCAGTATCTC |
| ACTB | CACCATTGGCAATGAGCGGTTC | AGGTCTTTGCGGATGTCCACGT |
