# Supplementary figures and images for: Single-cell transcriptome analysis reveals the metabolic changes and the prognostic value of malignant hepatocyte subpopulations and predict new therapeutic agents for hepatocellular carcinoma
Source: Front Oncol. 2023 Jan 31;13:1104262. doi: 10.3389/fonc.2023.1104262 (PMC9969971; doi:10.3389/fonc.2023.1104262)

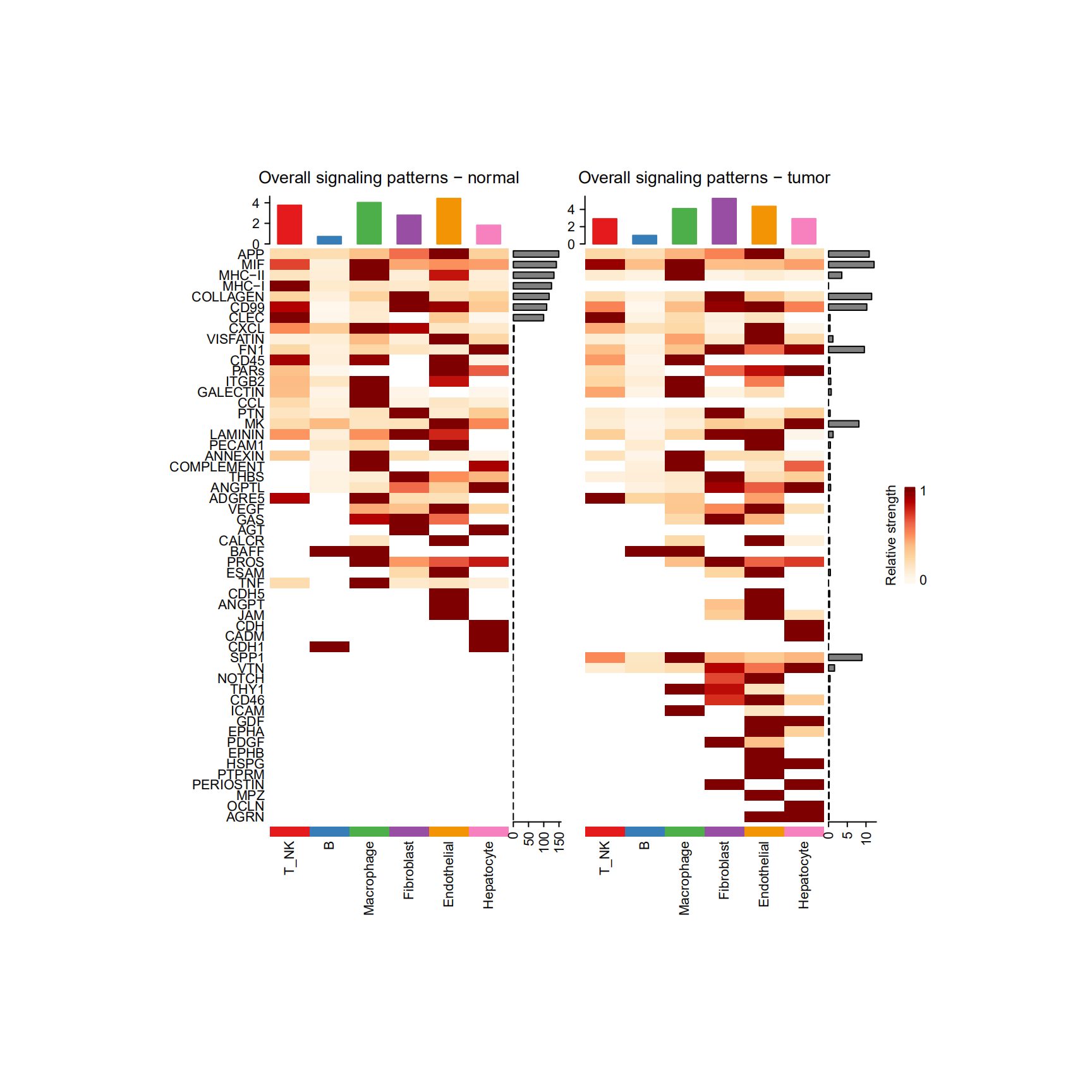

Supplement: Supplementary file 1 [file DataSheet_1.zip › Supplementary Material/Figure S1.TIF]

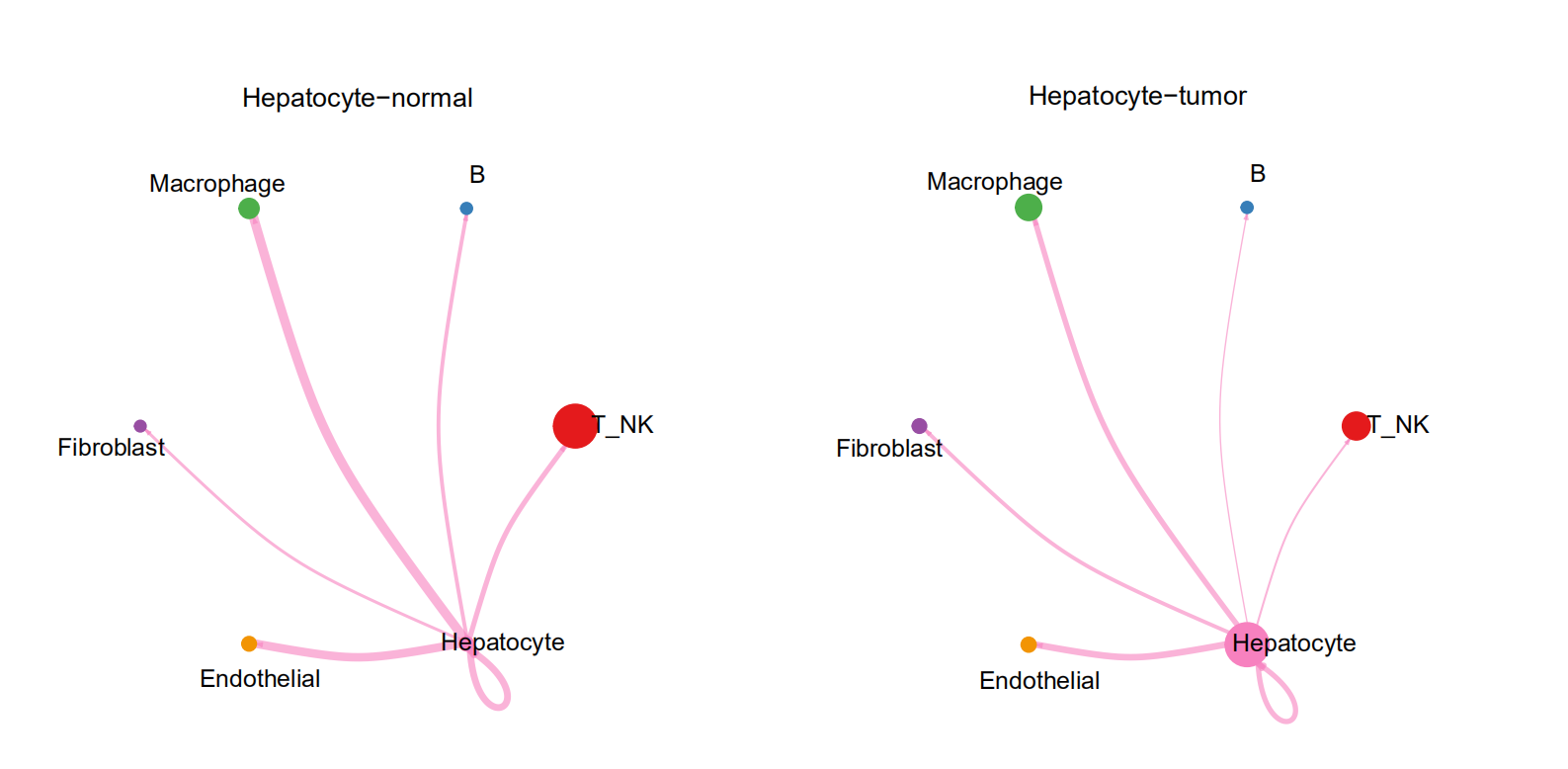

Supplement: Supplementary file 1 [file DataSheet_1.zip › Supplementary Material/Figure S2.TIF]

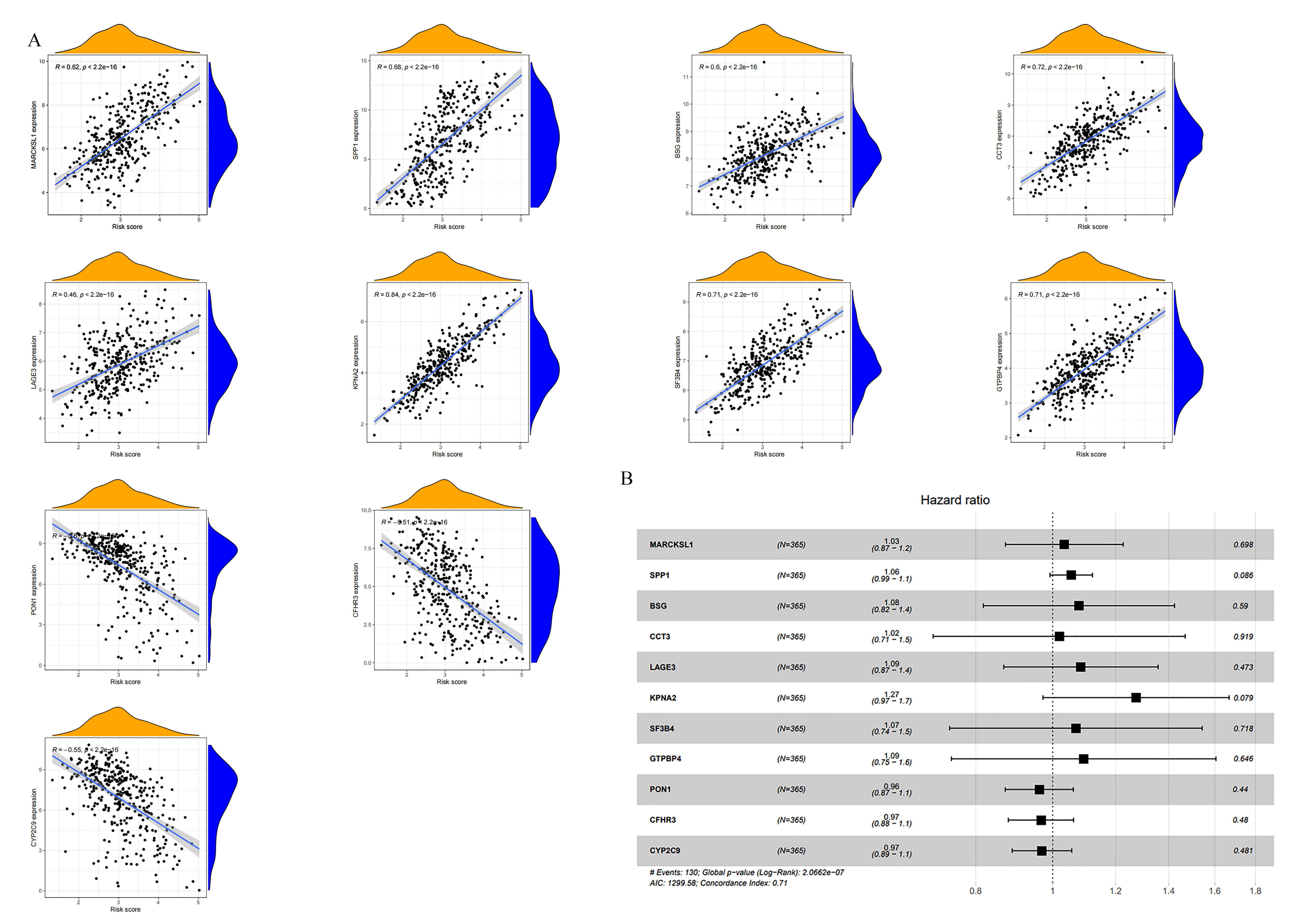

Supplement: Supplementary file 1 [file DataSheet_1.zip › Supplementary Material/Figure S3.jpg]
